# A Compend of the Practice of Medicine

**Published:** 1899-11

**Authors:** 


A Compend of the Practice of Medicine. By Daniel E. Hughes, M. D., Jefferson Medical College, Philadelphia. Sixth physician's edition. Thoroughly revised and enlarged. Including a section on Mental Diseases, and a very complete section on Skin Diseases. Pp., 625. Price, $2.25. Philadelphia: P. Blakiston's Sons & Co., 1012 Walnut St. 1899.

As stated on the title page, this book has reached its sixth edition. It has been enlarged and a chapter on mental diseases and one on skin diseases have been added. This book contains the essentials of medicine without entering into details.

Perhaps no work has been more popular for the purpose intended than has been this one. The author does not expect it to take the place of the more extensive text-books on practice, but it is to "aid the medical student at a time when practical demonstrations and ward classes were the exception in the college curriculum."

On page twenty-two, under the head of Typhoid Fever, the author says that the disease is characterized by "a constant lesion of Peyer's patches." This does not sound up to date. But the book is so admirably arranged and altogether so well gotten up it is recommended to any one needing a compend on general practice. B.

